# A comprehensive prognostic model for older patients with advanced non-small cell lung cancer receiving immunotherapy: a multicenter real-world study

**DOI:** 10.3389/fimmu.2026.1917659

**Published:** 2026-08-11

**Authors:** Xinyu Song, Juncai Lv, Yiming Qi, Hongying Zheng, Ziyuan Ren, Peilin Wang, Haiwang Sun, Hongbo Shen

**Affiliations:** 1 Department of Medical Oncology, Qilu Hospital, Cheeloo College of Medicine, Shandong University, Jinan, Shandong, China; 2 Department of Shandong Provincial Key Laboratory of Precision Oncology, Shandong Cancer Hospital and Institute, Shandong First Medical University and Shandong Academy of Medical Sciences, Jinan, Shandong, China; 3 Clinical Lab, The Affiliated Hospital of Qingdao University, Qingdao, Shandong, China

**Keywords:** comorbidity, immunotherapy, inflammation, non-small cell lung cancer, nutrition, older patients, prognostic model

## Abstract

**Objective:**

Older patients (aged ≥ 75 years) with advanced non-small cell lung cancer (NSCLC) receiving immunotherapy represent a growing but understudied population, yet few tools incorporate multidimensional host factors that influence prognosis. We therefore developed and externally validated a real-world prognostic model using bedside-available clinical variables, and compared its performance against conventional indices.

**Methods:**

This multicenter retrospective study included patients aged ≥ 75 years with advanced NSCLC receiving first-line immunotherapy. Cox regression analyses were performed to identify potential survival-associated host variables. A composite model, based on clinically relevant candidate variables identified through univariate screening, was developed using endpoint-specific L2-penalized Cox regression and internally validated with 1,000 bootstrap resamples. The final model was externally validated in two independent cohorts. Model performance was assessed using Harrell’s C-index, inverse probability of censoring-weighted (IPCW) cumulative/dynamic area under the curve (AUC), calibration, decision curve analysis, and Kaplan-Meier survival analysis with log-rank tests. The model was formally compared with conventional indices using paired bootstrap analyses.

**Results:**

A total of 241 patients were included, with 115 in the development cohort and 126 in the pooled external validation cohort. We developed the CALI (Comorbidity-Advanced Lung Cancer Inflammation Index) model integrating comorbidity burden (hypertension, diabetes, chronic obstructive pulmonary disease), recent weight loss, and inflammatory-nutritional status represented by ALI. In the development cohort, 1- and 2-year AUCs were 0.587 and 0.712 for overall survival (OS), 0.584 and 0.708 for progression-free survival (PFS), with significant stratification for OS (*P* = 0.013) and PFS (*P* = 0.021). In the pooled validation cohort, 1- and 2-year AUCs were 0.561 and 0.806 for OS, 0.553 and 0.793 for PFS, also significant stratification for OS (*P* = 0.006) and PFS (*P* = 0.008). In paired comparisons, CALI showed modest but significant OS improvements over conventional indices, whereas PFS gains were less consistent.

**Conclusion:**

CALI provided modest prognostic discrimination and risk stratification in older patients with advanced NSCLC receiving immunotherapy, with stronger and more consistent performance for OS than for PFS. The model offers a simple, bedside-applicable tool using routinely available clinical variables, and can be easily accessed via our online calculator (https://jccclv.github.io/CALI-calculator) for individualized risk stratification.

## Introduction

Immune checkpoint inhibitors (ICIs) have substantially reshaped the treatment landscape of advanced non-small cell lung cancer (NSCLC), leading to improved survival in selected patients (1, 2). However, the clinical trial evidence supporting these benefits comes predominantly from younger populations, limiting the generalizability of these findings to older patients. This gap is particularly concerning because lung cancer is an age-related malignancy, with a median age at diagnosis of approximately 71 years (3). As the global population continues to age, the number of older patients with advanced NSCLC is steadily increasing. Consequently, real-world data from this understudied population are urgently needed to inform clinical decision-making.

The survival benefits of immunotherapy have not been consistently observed in older patients (3, 4), and the overall benefit remains highly heterogeneous and less established than in the general NSCLC population (5). This discrepancy is largely driven by distinct biological and clinical features that distinguish older from younger patients, including immunosenescence, chronic low-grade inflammation, reduced nutritional reserve, comorbidities, as well as reduced treatment tolerance (6, 7). Together, these factors contribute to substantially greater host complexity and may influence immunotherapy response (8, 9). Thus, accurately characterizing host condition and identifying older patients likely to benefit from immunotherapy are important clinical priorities.

However, the prognostic tools currently available for older patients remain limited. Current prognostic research in immunotherapy has primarily focused on PD-L1 expression, inflammatory markers, and nutritional indices (10). For example, the widely used Advanced Lung Cancer Inflammation Index (ALI), calculated by body mass index (BMI) × serum albumin/neutrophil-to-lymphocyte ratio (NLR), has been associated with outcomes in advanced NSCLC. Other indices including NLR, Prognostic Nutritional Index (PNI), and platelet-to-lymphocyte ratio (PLR) have also been linked to prognosis (11–13). However, most existing studies have been conducted in unselected populations, with limited evidence specifically addressing older patients (14). Given that prognosis in older individuals is influenced not only by inflammation and nutrition but also by comorbidity burden, frailty, and treatment tolerance, these indices may not fully capture the complexity of host condition (15). Moreover, comorbidities are highly prevalent among older patients, and their cumulative comorbidity burden may affect long-term outcomes through systemic inflammation, nutritional reserve, organ function, and treatment tolerance (16–18).

Therefore, this study used multicenter real-world data to identify host-related factors associated with survival in older patients with advanced NSCLC receiving first-line immunotherapy, and to construct a composite prognostic model incorporating comorbidity burden, recent weight loss, and inflammatory-nutritional status, which captures key aspects of host status. The resulting model was developed in one cohort and externally validated in two independent cohorts, and may serve as a practical bedside tool for risk stratification in this understudied population.

## Materials and methods

### Study design and patient selection

**Figure 1:**
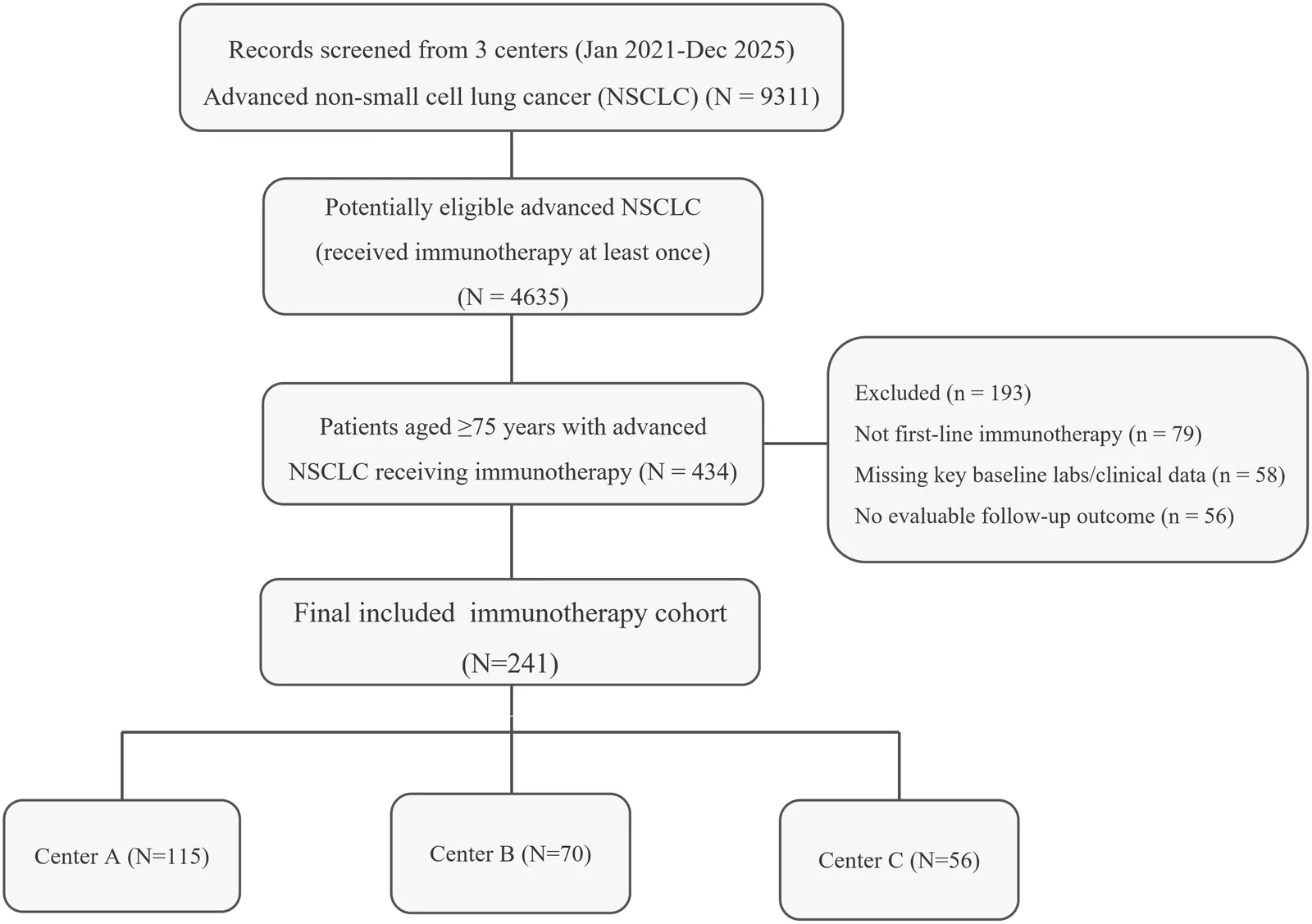
Flow diagram of the study design.

This multicenter retrospective study enrolled older patients (aged ≥75 years) with advanced NSCLC receiving first-line immunotherapy between January 2021 and December 2025 across three participating centers: Shandong Cancer Hospital (cohort A), Qilu Hospital of Shandong University (cohort B), and the Affiliated Hospital of Qingdao University (cohort C). Each has an independent patient catchment area and operates independently with its own clinical team and radiology review process. The inclusion criteria were: (1) age ≥ 75 years; (2) histologically or cytologically confirmed NSCLC; (3) stage IV disease (9th edition of the AJCC staging system); (4) receipt of first-line programmed cell death-1 (PD-1) or its ligand PD-L1 inhibitor-based therapy, including ICI monotherapy or immunotherapy combined with chemotherapy; and (5) availability of complete baseline clinical, laboratory, and follow-up data. The exclusion criteria were: (1) other active malignancies that could interfere with endpoint assessment; (2) receipt of immunotherapy outside the first-line setting; (3) substantial missing baseline or follow-up data. Cohort A served as the development cohort, while cohorts B and C served as independent external validation cohorts. This study was conducted in accordance with the Declaration of Helsinki and received ethical approval from the institutional review boards of all participating centers. The study flowchart is shown in **Figure 1**.

### Data collection and outcome assessment

Baseline variables included demographic characteristics, tumor features, comorbidities, and laboratory parameters. Candidate variables were selected based on clinical relevance and published literature. The study endpoints were overall survival (OS) and progression-free survival (PFS). OS was defined as the time from initiation of first-line immunotherapy to death from any cause or censoring at the last follow-up date. PFS was defined as the time from initiation of first-line immunotherapy to disease progression, death, or censoring at the date of last follow-up, whichever occurred first. Median follow-up time was estimated using the reverse Kaplan–Meier method and is reported together with the interquartile range (IQR). All survival times were calculated in days.

### Construction of the CALI model

To identify candidate variables for model construction, univariate Cox regression analyses were first performed separately for OS and PFS. Variables with *P* value< 0.05 in univariate analysis were considered candidate variables for potential model inclusion no matter in OS or PFS. Variables with P ≤ 0.10 in univariate analysis were entered into multivariate Cox regression as an exploratory step to assess independent contributions, but these results did not determine the final model composition, given the limited sample size and potential collinearity among host-related factors. The number of covariates in each multivariate model was restricted to no more than six based on univariate screening results and clinical relevance. The proportional hazards assumption was evaluated using Schoenfeld residuals.

For model development, binary variables were coded as 1 for presence and 0 for absence. All continuous variables were standardized using z-score transformation based on the mean and standard deviation of the development cohort, with the same parameters applied to the external validation cohorts. Endpoint-specific L2-penalized Cox proportional hazards models were developed for OS and PFS. The penalty parameters were selected in the development cohort using repeated 10-fold cross-validation and were fixed at 0.5 for OS and 1.0 for PFS. Each predictor contributed one degree of freedom; no interaction or spline terms were included. The linear predictor was calculated as:


$$\eta_{\text{i}}=\beta_{1}{\text{Z}}_{1\text{,i}}+\beta_{2}{\text{Z}}_{2\text{,i}}+\beta_{3}{\text{Z}}_{3\text{,i}}+\beta_{4}{\text{Z}}_{4\text{,i}}+\beta_{5}{\text{Z}}_{5\text{,i}}$$

where *β_1_* to *β_5_* represent the estimated Cox regression coefficients and *Z_1_* to *Z_5_* represent the standardized values of the selected variables. The risk score was calculated as:


$${Risk}_{i}=exp(\eta_{\text{i}})$$

with higher scores indicating greater predicted risk of death or disease progression. For external validation, the Cox regression coefficients and standardization parameters derived from the development cohort were applied without re-estimation. Patients were classified into high- and low-risk groups according to the median risk score in the development cohort. The coefficients and standardization parameters are provided in **Supplementary Table 1**.

### Model performance and external validation

External validation was performed in cohorts B and C using the fixed model coefficients and standardization parameters derived from the development cohort to calculate individual risk scores for OS and PFS.

Model performance was evaluated in terms of discrimination, calibration, clinical utility, and risk stratification. Discrimination was assessed using Harrell’s concordance index (C-index) and time-dependent receiver operating characteristic (ROC) analysis, with discrimination quantified by inverse probability of censoring-weighted (IPCW) cumulative/dynamic area under the curve (AUC) from 6 to 24 months. Pointwise 95% confidence interval (CI) for AUC estimates from 6 to 24 months were obtained using 1,000 bootstrap resamples, with the 12- and 24-month estimates reported separately. Formal comparisons at these two time points were based on paired bootstrap differences between CALI and each conventional index, with corresponding 95% confidence intervals and two-sided P values. Calibration was assessed separately for OS and PFS at 1- and 2-year in the development cohort and the pooled validation cohort by comparing predicted event probabilities with Kaplan-Meier-estimated observed event probabilities. Clinical utility was evaluated by decision curve analysis (DCA).

Model complexity was assessed using events per variable (EPV), calculated separately for OS and PFS by dividing the number of endpoint events in the development cohort by the number of predictors in the final model. Internal validation was performed separately for OS and PFS using 1,000 bootstrap resamples of the development cohort. Within each bootstrap sample, standardization parameters were re-estimated and the final L2-penalized Cox model was refitted using the fixed predictor set and the cross-validation-selected penalty parameter. Each bootstrap-fitted model was evaluated in both the bootstrap sample and the original development cohort. Optimism was defined as the difference between bootstrap-sample and original-cohort performance, and optimism-corrected performance was calculated by subtracting mean optimism from apparent performance. Internal validation assessed Harrell’s C-index and calibration slope, with 95% confidence intervals derived from the bootstrap distribution of the corrected estimates.

For risk stratification, patients in the validation cohorts were categorized into high- and low-risk groups using the median risk score derived from the development cohort. Kaplan-Meier curves were generated for OS and PFS, and differences between groups were compared using the log-rank test.

To formally compare discriminative performance, Harrell’s C-indices of CALI and conventional indices (ALI, PNI, NLR, and PLR) were estimated separately for OS and PFS in cohort A and the pooled validation cohort (B and C). Paired differences in C-index, calculated as CALI minus the comparator, together with 95% confidence intervals and two-sided P values, were obtained using 1,000 paired bootstrap resamples. The Benjamini-Hochberg procedure was applied within each cohort-endpoint family for C-index and AUC comparisons to control the false discovery rate across the four comparisons.

### Statistical analysis

All statistical analyses were performed using SPSS (version 29.0, IBM Corp., Armonk, NY, USA) and Python (version 3.13.9). Survival analyses and concordance indices were calculated using the lifelines package (version 0.30.3), and time-dependent ROC and DCA analyses were conducted using R (version 4.3.0) with the rmda package. Categorical variables are presented as frequencies and percentages, and continuous variables as means ± SD or medians with interquartile ranges. Differences between the development and validation cohorts were assessed using Pearson’s chi-square test for categorical variables and the Mann-Whitney U test or Student’s t-test for continuous variables. Missing data were handled by complete-case analysis. All statistical tests were two-tailed, and a *P* value<0.05 was considered statistically significant.

## Results

### Baseline characteristics of older patients receiving immunotherapy

A total of 241 older patients with advanced NSCLC receiving immunotherapy were included, comprising 115 patients in the development cohort (cohort A) and 126 patients in the validation cohorts (cohorts B and C). The median follow-up time was 14.0 months (IQR, 6.3-35.2) in cohort A, 16.4 months (IQR, 7.4-34.0) in cohort B, and 13.5 months (IQR, 7.9-19.4) in cohort C. During follow-up, 47 OS events (40.9%) and 69 PFS events (60.0%) occurred in cohort A, 25 OS events (35.7%) and 32 PFS events (45.7%) occurred in cohort B, and 21 OS events (37.5%) and 29 PFS events (51.8%) occurred in cohort C.

In the development cohort A, the mean age was 77.71 ± 3.01 years, and 87 patients (75.7%) were male. Most patients had an Eastern Cooperative Oncology Group (ECOG) performance status of 0-1 (87.8%). The majority of patients received ICI plus chemotherapy (79.1%), while 20.9% received ICI monotherapy. Adenocarcinoma and squamous cell carcinoma accounted for 52.2% and 43.5% of cases, respectively. The pooled validation cohort B+C had similar baseline characteristics, with a mean age of 77.43 ± 2.34 years, 69.0% male, an ECOG 0–1 rate of 88.9%, treatment with ICI plus chemotherapy and ICI monotherapy in 75.4% and 24.6% of patients, and adenocarcinoma and squamous cell carcinoma accounting for 46.8% and 45.2% of cases, respectively. Baseline characteristics were generally comparable between the two cohorts (**Table 1**).

**Table 1:** Baseline characteristics of patients receiving immunotherapy.

Variable	Cohort A (n = 115)	Cohorts B+C (n = 126)	P value
Age (years)	77.71 ± 3.01	77.43 ± 2.34	0.417
Sex			0.253
Male	87 (75.7%)	87 (69.0%)	
Female	28 (24.3%)	39 (31.0%)	
ECOG PS (0–1 vs ≥2)			0.797
0–1	101 (87.8%)	112 (88.9%)	
≥2	14 (12.2%)	14 (11.1%)	
BMI (kg/m²)	23.36 ± 3.37	23.66 ± 2.45	0.434
Treatment regimen			0.490
ICI monotherapy	24 (20.9%)	31 (24.6%)	
ICI plus chemotherapy	91 (79.1%)	95 (75.4%)	
Weight loss ≥5% within 3 months			0.716
Yes	26 (22.6%)	31 (24.6%)	
No	89 (77.4%)	95 (75.4%)	
Histology			0.442
Adenocarcinoma	60 (52.2%)	59 (46.8%)	
Squamous	50 (43.5%)	57 (45.2%)	
Other	5 (4.3%)	10 (7.9%)	
Smoking history			0.972
Yes	76 (66.1%)	83 (65.9%)	
No	39 (33.9%)	43 (34.1%)	
Cardiac disease			0.294
Yes	17 (14.8%)	13 (10.3%)	
No	98 (85.2%)	113 (89.7%)	
Diabetes			0.622
Yes	21 (18.3%)	20 (15.9%)	
No	94 (81.7%)	106 (84.1%)	
Hypertension			0.675
Yes	45 (39.1%)	46 (36.5%)	
No	70 (60.9%)	80 (63.5%)	
COPD			0.463
Yes	64 (55.7%)	76 (60.3%)	
No	51 (44.3%)	50 (39.7%)	
CKD			0.050
Yes	4 (3.5%)	0 (0.0%)	
No	111 (96.5%)	126 (100.0%)	
Pneumonia			0.834
Yes	25 (21.7%)	26 (20.6%)	
No	90 (78.3%)	100 (79.4%)	
Lung metastasis			0.727
Yes	61 (53.0%)	64 (50.8%)	
No	54 (47.0%)	62 (49.2%)	
Bone metastasis			0.880
Yes	50 (43.5%)	56 (44.4%)	
No	65 (56.5%)	70 (55.6%)	
Brain metastasis			0.145
Yes	26 (22.6%)	39 (31.0%)	
No	89 (77.4%)	87 (69.0%)	
Liver metastasis			0.503
Yes	23 (20.0%)	21 (16.7%)	
No	92 (80.0%)	105 (83.3%)	

BMI, body mass index; CKD, chronic kidney disease; COPD, chronic obstructive pulmonary disease; ECOG PS, Eastern Cooperative Oncology Group performance status.

### Performance of conventional indices and prognostic factors screening

**Figure 2:**
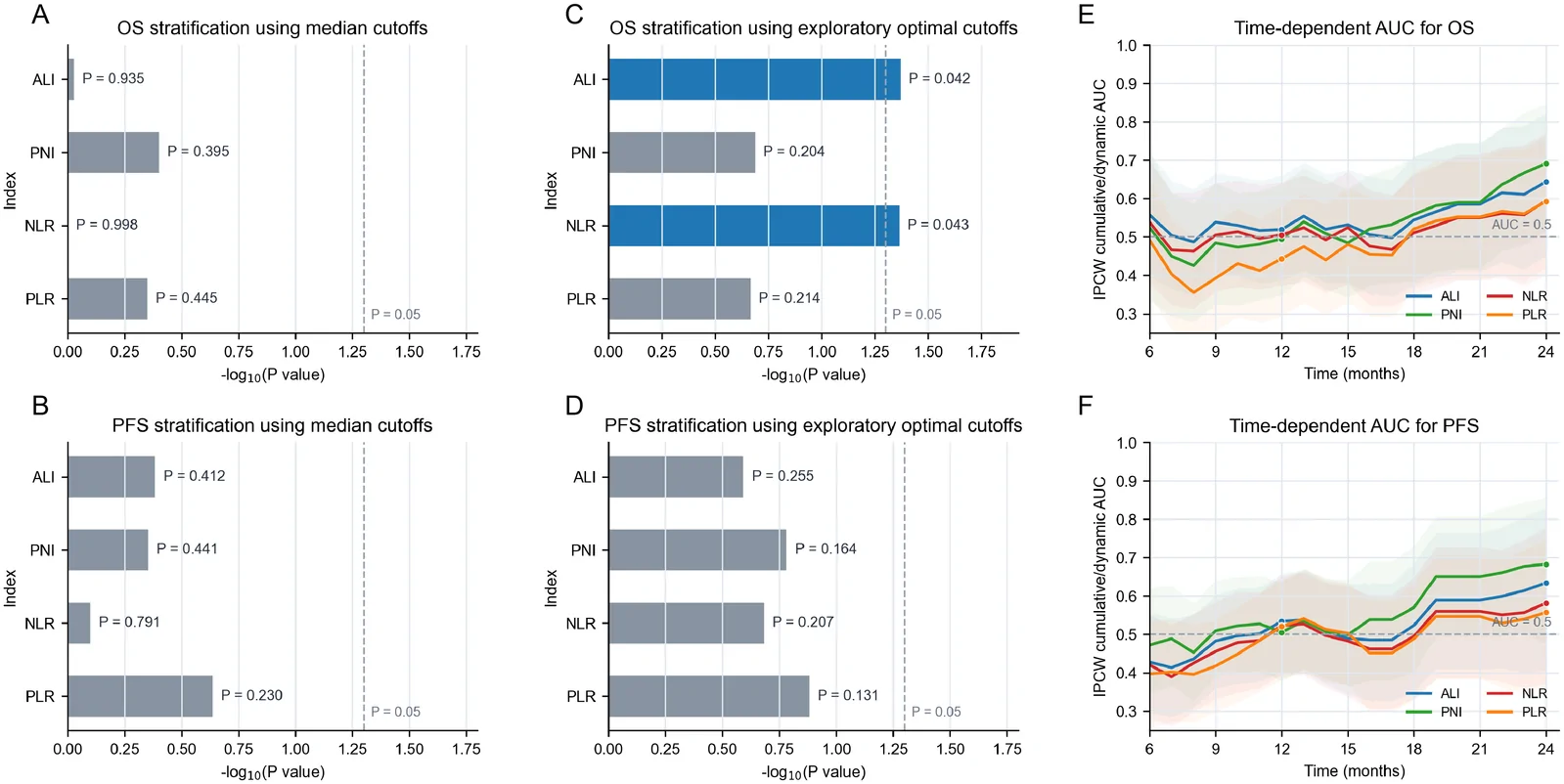
Comparative prognostic performance of conventional inflammatory and nutritional indices in older patients with advanced NSCLC receiving immunotherapy. **(A, B)** Log-rank P values for OS **(A)** and PFS **(B)** using median cutoff values. **(C, D)** Log-rank P values for OS **(C)** and PFS **(D)** stratification using optimal cutoff values. **(E, F)** Inverse probability of censoring-weighted (IPCW) cumulative/dynamic AUC curves from 6 to 24 months for OS **(E)** and PFS **(F)**. Shaded areas represent pointwise 95% confidence intervals based on 1,000 bootstrap resamples, and markers indicate the 12- and 24-month estimates. The horizontal dashed line denotes an AUC of 0.5.

To assess whether conventional indices remain applicable in the older population, we first evaluated the performance of ALI, PNI, NLR, and PLR in the development cohort. However, none of these indices showed significant stratification using median cutoff values in both OS and PFS (**Figures 2A, B**; **Supplementary Figure 1**). When optimal cutoffs were applied, ALI (*P* = 0.042) and NLR (*P* = 0.043) achieved significant OS stratification (**Figures 2C, D**; **Supplementary Figure 2**). Time-dependent ROC analysis showed limited discriminative ability for all these conventional indices, with AUCs close to 0.5 at 1 year and remaining below 0.7 at 2 years for both OS and PFS (**Figures 2E, F**). These findings suggest that conventional indices alone are insufficient for reliable risk stratification in older patients receiving immunotherapy.

Although ALI and NLR showed stratification signal at optimal cutoffs, their overall discriminative ability remained limited, suggesting the above conventional indices may not sufficiently capture the complex host condition of older patients. To identify additional host-related factors, we performed univariate and multivariate Cox regression analyses in the development cohort. For OS, hypertension (HR = 1.895; 95% CI, 1.098-3.272; *P* = 0.022), chronic obstructive pulmonary disease (COPD) (HR = 1.943; 95% CI, 1.066-3.542; *P* = 0.030), weight loss ≥ 5% within 3 months (HR = 2.099; 95% CI, 1.130-3.896; *P* = 0.019), and higher BMI (HR = 0.907; 95% CI, 0.834-0.986; *P* = 0.022) showed significant associations with survival. In multivariate analysis, none of these variables remained statistically significant (**Table 2**; **Supplementary Figure 3**). For PFS, diabetes (HR = 1.774; 95% CI, 1.004-3.134; *P* = 0.048), hypertension (HR = 1.680; 95% CI, 1.051-2.684; *P* = 0.030), and higher BMI (HR = 0.914; 95% CI, 0.853-0.980; *P* = 0.011) showed significant associations with survival. In multivariate analysis, diabetes (HR = 1.960; 95% CI, 1.074-3.575; *P* = 0.028) and hypertension (HR = 1.629; 95% CI, 1.016-2.610; *P* = 0.043) remained independently associated with PFS. Adenosine deaminase (ADA) was also significant (HR = 0.538; 95% CI, 0.294-0.984; *P* = 0.044) but lacked signal in OS (**Table 3**; **Supplementary Figure 4**).

**Table 2:** Univariate and multivariate analyses of factors associated with OS.

Variable	Univariate HR (95% CI)	*P*	Multivariate HR (95% CI)	*P*
Sex (male vs female)	1.026 (0.555 – 1.899)	0.934	—	—
ECOG (0–1 vs ≥ 2)	1.426 (0.608 – 3.344)	0.415	—	—
BMI (kg/m²; per 1 unit)	0.907 (0.834 – 0.986)	0.022	0.925 (0.843 – 1.015)	0.099
Weight loss ≥ 5% in 3 months (yes vs no)	2.099 (1.130 – 3.896)	0.019	1.497 (0.779 – 2.874)	0.226
Histology (squamous vs adenocarcinoma)	0.891 (0.515 – 1.540)	0.678	—	—
Smoking history (yes vs no)	1.363 (0.746 – 2.491)	0.314	—	—
Cardiac disease (yes vs no)	1.699 (0.852 – 3.386)	0.132	—	—
Diabetes (yes vs no)	1.826 (0.937 – 3.557)	0.077	1.613 (0.778 – 3.344)	0.199
Hypertension (yes vs no)	1.895 (1.098 – 3.272)	0.022	1.362 (0.742 – 2.499)	0.319
COPD (yes vs no)	1.943 (1.066 – 3.542)	0.030	1.484 (0.740 – 2.975)	0.266
CKD (yes vs no)	1.154 (0.357 – 3.723)	0.811	—	—
Pneumonia (yes vs no)	1.666 (0.931 – 2.981)	0.086	—	—
Lung metastasis (yes vs no)	0.754 (0.434 – 1.309)	0.316	—	—
Bone metastasis (yes vs no)	0.894 (0.512 – 1.560)	0.693	—	—
Brain metastasis (yes vs no)	0.996 (0.539 – 1.839)	0.990	—	—
Liver metastasis (yes vs no)	1.125 (0.575 – 2.199)	0.731	—	—
log1p [WBC (10^9^/L)]	1.171 (0.499 – 2.746)	0.717	—	—
RBC (10^12^/L)	0.677 (0.411 – 1.113)	0.124	—	—
log1p [Platelet (10^9^/L)]	1.201 (0.597 – 2.416)	0.607	—	—
log1p [Neutrophils (10^9^/L)]	1.198 (0.575 – 2.493)	0.629	—	—
ALC (10^9^/L)	1.002 (0.611 – 1.646)	0.992	—	—
AMC (10^9^/L)	0.972 (0.321 – 2.947)	0.960	—	—
Albumin	0.942 (0.871 – 1.019)	0.139	—	—
log1p (LDH)	1.803 (0.974 – 3.338)	0.060	1.675 (0.775 – 3.621)	0.190
log1p (ADA)	0.641 (0.294 – 1.398)	0.263	—	—
log1p [ALI (per 1 unit)]	0.745 (0.479 – 1.160)	0.192	—	—
log1p (NLR)	1.244 (0.653 – 2.369)	0.506	—	—
log1p (PLR)	1.213 (0.675 – 2.179)	0.518	—	—
log1p (LMR)	0.958 (0.469 – 1.958)	0.907	—	—
log1p (SII)	1.150 (0.792 – 1.670)	0.463	—	—
log1p (SIRI)	1.089 (0.630 – 1.884)	0.760	—	—
PNI	0.969 (0.914 – 1.026)	0.280	—	—

ADA, Adenosine Deaminase; ALC, Absolute Lymphocyte Count; ALI, Advanced Lung Cancer Inflammation Index; AMC, Absolute Monocyte Count; BMI, Body Mass Index; CEA, Carcinoembryonic Antigen; CI, Confidence Interval; CKD, Chronic Kidney Disease; COPD, Chronic Obstructive Pulmonary Disease; ECOG, Eastern Cooperative Oncology Group; HR, Hazard Ratio; LDH, Lactate Dehydrogenase; LMR, Lymphocyte- to- Monocyte Ratio; NLR, Neutrophil-to-Lymphocyte Ratio; PLR, Platelet-to-Lymphocyte Ratio; PNI, Prognostic Nutritional Index; RBC, Red Blood Cell; SII, Systemic Inflammatory Index; SIRI, Systemic Inflammatory Response Index; WBC, White Blood Cell.

**Table 3:** Univariate and multivariate analyses of factors associated with PFS.

Variable	Univariate HR (95% CI)	*P*	Multivariate HR (95% CI)	*P*
Sex (male vs female)	1.171 (0.694 – 1.975)	0.553	—	—
ECOG (0–1 vs ≥ 2)	1.525 (0.782 – 2.977)	0.216	—	—
BMI (kg/m²; per 1 unit)	0.914 (0.853 – 0.980)	0.011	0.931 (0.864 – 1.003)	0.060
Weight loss ≥ 5% in 3 months (yes vs no)	1.564 (0.908 – 2.695)	0.107	—	—
Histology (squamous vs adenocarcinoma)	0.847 (0.533 – 1.346)	0.482	—	—
Smoking history (yes vs no)	1.354 (0.825 – 2.224)	0.231	—	—
Cardiac disease (yes vs no)	1.669 (0.851 – 3.274)	0.136	—	—
Diabetes (yes vs no)	1.774 (1.004 – 3.134)	0.048	1.960 (1.074 – 3.575)	0.028
Hypertension (yes vs no)	1.680 (1.051 – 2.684)	0.030	1.629 (1.016 – 2.610)	0.043
COPD (yes vs no)	1.460 (0.902 – 2.363)	0.123	—	—
CKD (yes vs no)	0.719 (0.225 – 2.300)	0.579	—	—
Pneumonia (yes vs no)	1.230 (0.737 – 2.053)	0.428	—	—
Lung metastasis (yes vs no)	0.782 (0.490 – 1.248)	0.303	—	—
Bone metastasis (yes vs no)	1.085 (0.680 – 1.729)	0.733	—	—
Brain metastasis (yes vs no)	1.163 (0.700 – 1.931)	0.561	—	—
Liver metastasis (yes vs no)	1.407 (0.819 – 2.416)	0.216	—	—
log1p [WBC (10^9^/L)]	0.737 (0.349 – 1.556)	0.423	—	—
RBC (10^12^/L)	0.684 (0.455 – 1.028)	0.068	0.745 (0.475 – 1.168)	0.199
log1p [Platelet (10^9^/L)]	0.846 (0.458 – 1.562)	0.592	—	—
log1p [Neutrophils (10^9^/L)]	0.805 (0.423 – 1.534)	0.510	—	—
ALC (109/L)	0.833 (0.543 – 1.278)	0.402	—	—
AMC (10^9/L)	0.612 (0.242 – 1.548)	0.300	—	—
Albumin	0.952 (0.891 – 1.016)	0.139	—	—
log1p (LDH)	1.529 (0.899 – 2.602)	0.117	—	—
log1p (ADA)	0.564 (0.299 – 1.064)	0.077	0.538 (0.294–0.984)	0.044
log1p [ALI (per 1 unit)]	0.814 (0.557 – 1.189)	0.287	—	—
log1p (NLR)	1.122 (0.646 – 1.950)	0.682	—	—
log1p (PLR)	1.125 (0.702 – 1.804)	0.624	—	—
log1p (LMR)	0.946 (0.527 – 1.697)	0.853	—	—
log1p (SII)	0.987 (0.721 – 1.351)	0.936	—	—
log1p (SIRI)	0.943 (0.591 – 1.502)	0.804	—	—
PNI	0.960 (0.914–1.009)	0.108	—	—

Same as **Table 2**.

Based on the above findings, hypertension, diabetes, COPD, and weight loss were selected for their statistical significance in univariate analysis. ADA was excluded because it was only observed in multivariate analysis but lacked survival association in univariate analysis. As for ALI, it was included because our preliminary comparison of conventional indices showed that ALI and NLR had OS stratification signals at their optimal cutoffs, while BMI showed statistical significance in univariate analysis; given that ALI is a clinically established composite indicator that already integrates both BMI and NLR, we retained ALI directly without separately entering BMI. Thus, ALI, hypertension, diabetes, COPD, and weight loss were selected as candidate variables for the model.

### Development and performance of the CALI model

Using the above candidate variables, we constructed a composite model that combines the established inflammatory-nutritional index ALI with common comorbidities in older patients, so we named it the CALI (Comorbidity-Advanced Lung Cancer Inflammation Index) model. Bootstrap internal validation showed the apparent C-index was 0.673 for OS and 0.609 for PFS, with optimism-corrected values of 0.649 (95% CI, 0.561-0.741) and 0.578 (95% CI, 0.508-0.660), respectively. The optimism-corrected calibration slopes were 1.502 (95% CI, 0.643-2.467) for OS and 1.572 (95% CI, 0.349-2.870) for PFS (**Supplementary Table 2**).

**Figure 3:**
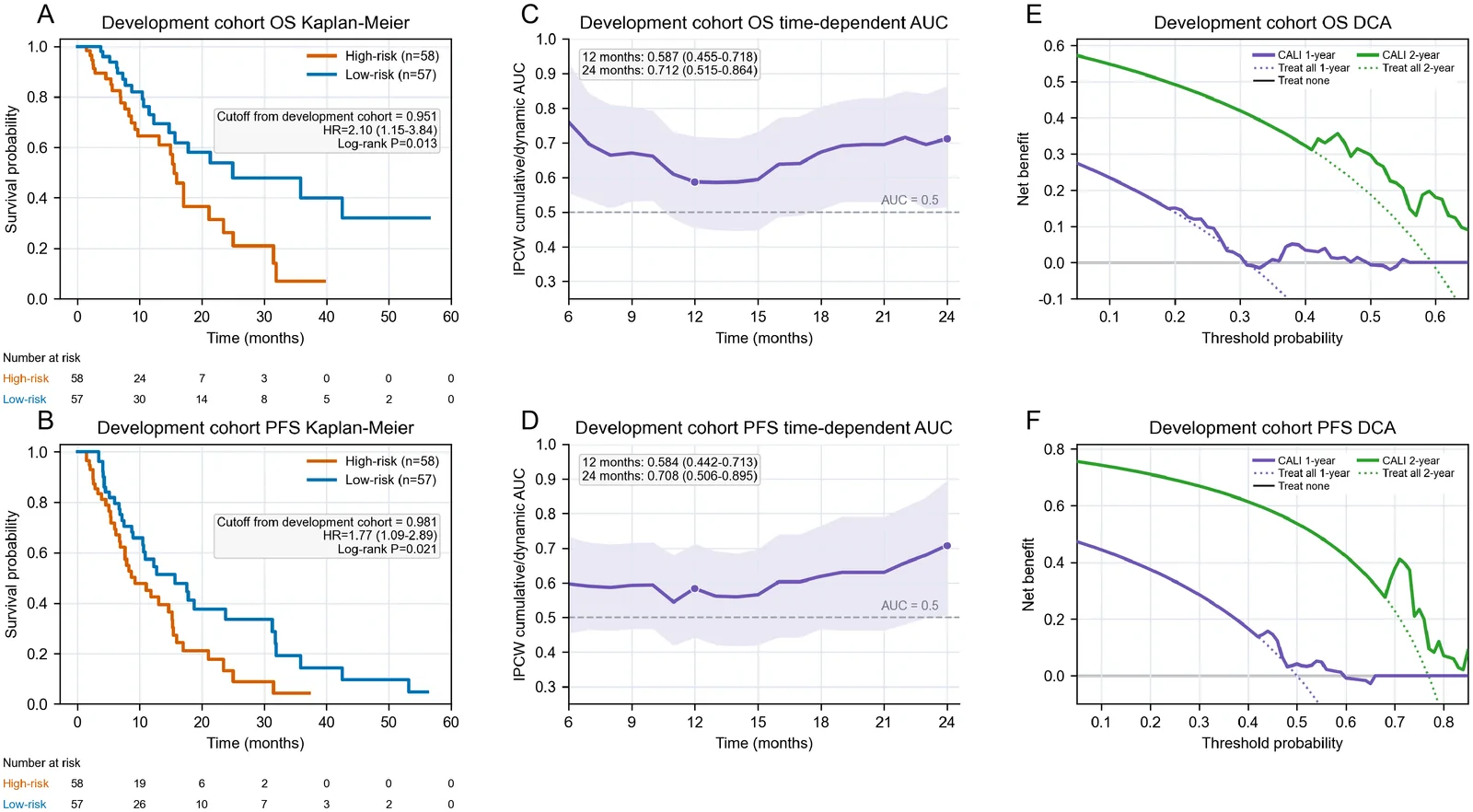
Development and prognostic performance of the CALI model in the development cohort. **(A, B)** Kaplan-Meier survival curves for OS **(A)** and PFS **(B)** stratified by the median CALI risk score. **(C, D)** IPCW cumulative/dynamic AUC curves from 6 to 24 months for OS **(C)** and PFS **(D)**. **(E, F)** Decision curve analysis (DCA) for OS **(E)** and PFS **(F)**.

In the development cohort, the CALI model demonstrated effective prognostic stratification. Kaplan-Meier analysis based on the median model-derived risk score showed significant separation for OS (HR = 2.10; 95% CI, 1.15-3.84; log-rank *P* = 0.013) (**Figure 3A**). For PFS, the model also achieved statistically significant risk stratification (HR = 1.77; 95% CI, 1.09-2.89; log-rank *P* = 0.021) (**Figure 3B**). IPCW cumulative/dynamic AUC analysis was performed from 6 to 24 months. For OS, the 1- and 2-year AUCs were 0.587 (95% CI, 0.455-0.718) and 0.712 (95% CI, 0.515-0.864), respectively. For PFS, the corresponding AUCs were 0.584 (95% CI, 0.442-0.713) and 0.708 (95% CI, 0.506-0.895), respectively (**Figures 3C, D**). DCA likewise suggested favorable net benefit across a range of threshold probabilities (**Figures 3E, F**).

### External validation of the CALI model

**Figure 4:**
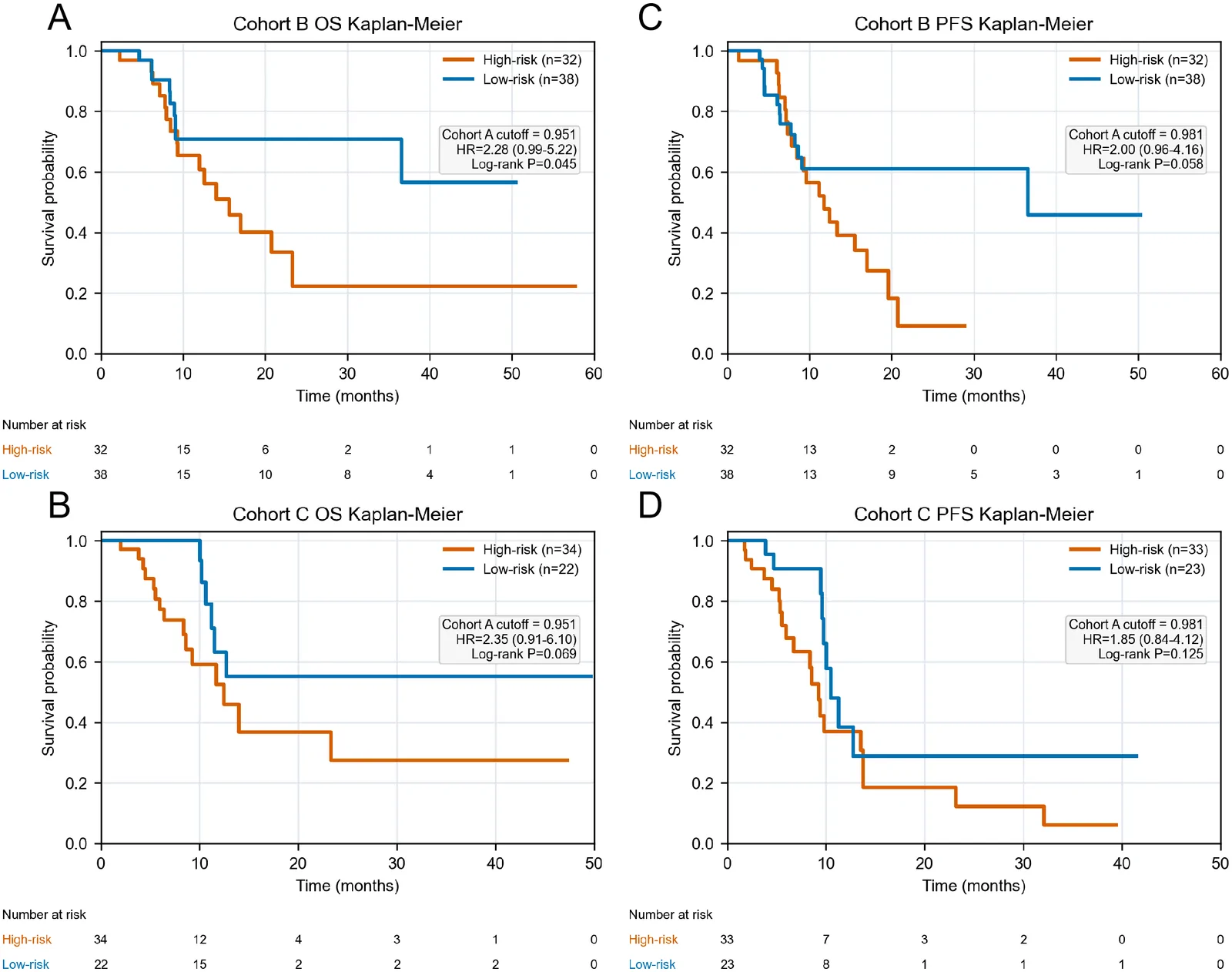
Kaplan-Meier survival analysis of the CALI model in external validation cohorts. **(A, B)** Kaplan-Meier curves for OS in cohort B **(A)** and cohort C **(B)**. **(C, D)** Kaplan-Meier curves for PFS in cohort B **(C)** and cohort C **(D)**. Patients were stratified into high- and low-risk groups using the endpoint-specific median risk-score cutoffs derived from the development cohort. P values were calculated using the log-rank test.

We next validated the CALI model in two independent external cohorts using the prespecified risk-score cutoffs derived from the development cohort. In cohort B, the CALI model significantly stratified OS (log-rank *P* = 0.045), with a similar trend observed in cohort C (log-rank *P* = 0.069). For PFS, the direction of effect was consistent in both cohorts, although statistical significance was not reached (log-rank *P* = 0.058 and 0.125, respectively) (**Figure 4**). Given the limited sample sizes of the validation cohorts, we pooled cohorts B and C to improve statistical power.

**Figure 5:**
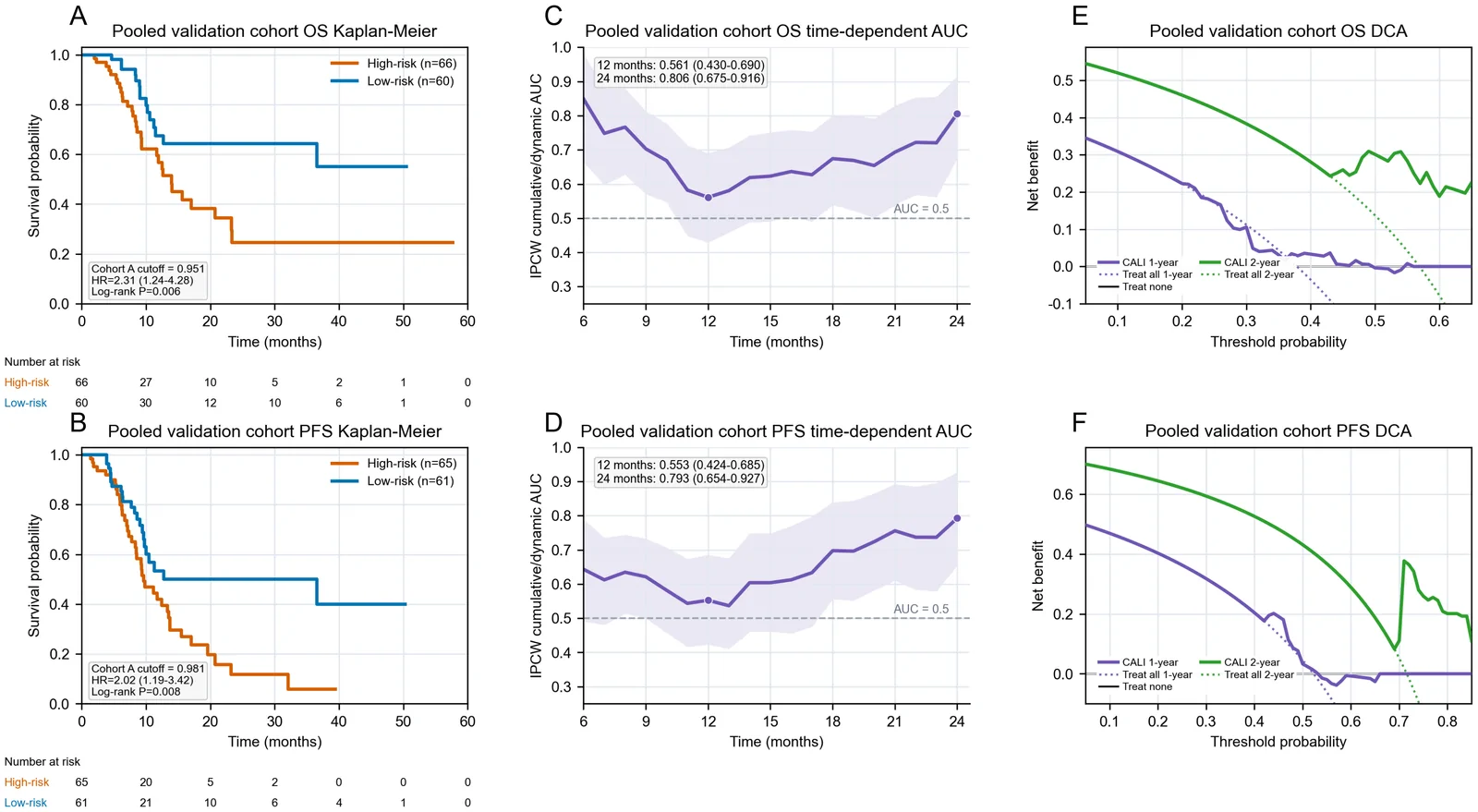
External validation of the CALI model in the pooled validation cohorts. **(A, B)** Kaplan-Meier curves for OS **(A)** and PFS **(B)** stratified by the prespecified endpoint-specific cutoffs derived from the development cohort. **(C, D)** Time-dependent ROC curves for OS **(C)** and PFS **(D)**. **(E, F)** DCA for OS **(E)** and PFS **(F)**.

When cohorts B and C were pooled, Kaplan-Meier analysis showed significant differences in OS (HR = 2.31; 95% CI, 1.24-4.28; log-rank *P* = 0.006) (**Figure 5A**) and PFS (HR = 2.02; 95% CI, 1.19-3.42; log-rank *P* = 0.008) (**Figure 5B**) between the two risk groups. IPCW cumulative/dynamic AUC analysis showed 1- and 2-year AUCs of 0.561 (95% CI, 0.430-0.690) and 0.806 (95% CI, 0.675-0.916) for OS (**Figure 5C**), and 0.553 (95% CI, 0.424-0.685) and 0.793 (95% CI, 0.654-0.927) for PFS (**Figure 5D**), respectively. DCA also suggested favorable net benefit across a range of threshold probabilities (**Figures 5E, F**). These findings indicate that the CALI model effectively stratifies prognosis in the pooled external validation cohort, particularly for long-term survival.

To further assess the incremental value of CALI over conventional indices, we compared their discriminative performance using time-dependent AUC curves and formal paired C-index comparisons. In paired time-dependent AUC comparisons, no significant differences were observed in the development cohort either in OS or PFS at 1 or 2 years. In the pooled validation cohort, only at 2 years CALI showed significantly higher OS AUCs than all four conventional indices and higher PFS AUCs than ALI, NLR, and PLR, whereas the difference versus PNI was not statistically significant (**Supplementary Table 3**). In formal paired C-index comparisons, CALI provided modest improvements in OS discrimination over ALI, PNI, NLR, and PLR in both development and the combined validation cohorts (all FDR-adjusted *P* < 0.05). For PFS, the improvements were less consistent. CALI significantly outperformed all indices in the development cohort except PNI, whereas in the combined validation cohort, only the comparison with PLR remained significant after FDR correction (FDR-adjusted *P* = 0.032) (**Supplementary Table 4**). The corresponding AUC trajectories are shown in **Supplementary Figure 5**. Overall, the incremental discriminatory performance of CALI was more consistent for OS than for PFS.

Calibration curves for the CALI model showed generally acceptable agreement between predicted and observed probabilities for both OS and PFS in the development and the pooled validation cohorts (**Supplementary Figure 6**).

### Development of the web-based calculator

To facilitate clinical application of the CALI model, we developed a web-based calculator which is available at https://jccclv.github.io/CALI-calculator. After entering routinely available baseline variables (height, weight, serum albumin, neutrophil count, lymphocyte count; whether have hypertension, diabetes, COPD, and weight loss ≥5% within 3 months), the calculator automatically computes CALI risk scores and classifies patients into high- or low-risk groups. All calculations are performed locally without uploading or storing patient data.

## Discussion

In this multicenter real-world study, we developed and externally validated the CALI model, a composite prognostic tool for patients aged ≥75 years with advanced NSCLC receiving immunotherapy, integrating comorbidity burden (hypertension, diabetes, and COPD), recent weight loss, and inflammatory-nutritional status (represented by ALI) into a unified framework. In paired C-index comparisons, CALI provided modest but significant improvements in OS discrimination compared with conventional indices in both the development and combined external validation cohorts. By capturing multidimensional host factors that are routinely available in clinical practice, the CALI model offers a practical, bedside-applicable tool for risk stratification in this understudied population.

Although several inflammatory and nutritional indices have been associated with prognosis in NSCLC patients receiving immunotherapy, their predictive value in older populations remains limited. The ALI has been shown to be associated with outcomes in advanced NSCLC (12), but our results suggest that ALI-based stratification using median cutoff failed to distinguish OS or PFS. Similarly, other commonly used indices including PNI, NLR, and PLR have shown prognostic utility in NSCLC immunotherapy (2, 5, 19), yet they each capture only one dimension of host status. PNI reflects nutritional and immune status, NLR captures systemic inflammation, and PLR is primarily a marker of platelet-mediated inflammation. Other complex composite scores including Lung Immune Prognostic Index (LIPI, based on derived NLR and LDH) (20), the modified Glasgow Prognostic Score (mGPS, based on CRP and albumin) (21), the Controlling Nutritional Status score (CONUT, based on albumin, lymphocyte count, and cholesterol) (22), and the Systemic Immune-Inflammation Index (SII, calculated as platelet × neutrophil/lymphocyte) (23), have also been studied in this setting, but they similarly focus on selected parameters without incorporating comorbidity burden, a key determinant of prognosis in older patients (24). Collectively, these observations suggest that reliance on any single or narrowly focused index may be insufficient to capture the full complexity of host condition in older patients, highlighting the need for a more comprehensive, multidomain prognostic tool for this population.

Each component of the CALI model has biological and clinical rationale. Hypertension is associated with chronic low-grade inflammation and vascular dysfunction, which may impair immune cell trafficking and antitumor immunity (25). Diabetes mellitus has been linked to poorer ICI outcomes in NSCLC (26), likely through an immunosuppressive tumor microenvironment and attenuated T-cell immune responses (27). COPD, while associated with systemic inflammation and immune dysregulation (28, 29), has also been linked to improved outcomes in some ICI-treated cohorts (30), underscoring the context-dependent nature of comorbidity effects on immunotherapy. Recent weight loss captures the dynamic aspect of nutritional decline, which ALI alone does not fully reflect. By integrating these three domains including comorbidity burden, recent weight loss, and inflammatory-nutritional status, the CALI model provides a more comprehensive assessment of host condition in older patients. This biological grounding likely contributes to its stronger performance for OS than for PFS.

However, methodological factors inherent to PFS assessment may also play a substantial role. First, PFS assessment in retrospective studies is subject to inherent biases, including variability in imaging intervals, subjective interpretation of progression, and the influence of clinical decision-making. These factors are particularly pronounced in older patients, who often have irregular follow-up due to reduced mobility or competing health priorities. Second, treatment discontinuation due to toxicity or patient preference may obscure true progression events (31), and this is especially common in older patients with reduced tolerance. Third, OS captures the cumulative impact of comorbidity burden, frailty, and nutritional decline, which may be more relevant to long-term survival than to short-term disease control (32). Forth, the relatively shorter follow-up in validation cohort C may have further limited PFS event accrual and statistical power for individual cohort analysis, which was overcome when cohorts B and C were pooled. Together, these considerations support the greater reliability of OS as an endpoint in older populations and explain why the predictive utility was more evident for OS than for PFS.

Nevertheless, several limitations should also be acknowledged. First, the retrospective design inevitably introduced selection bias, as data were collected from existing medical records without standardized protocols for all variables. Second, although external validation was performed in two independent cohorts, the sample size of individual cohorts were relatively small, and all three participating centers are located within the same province, which may limit the generalizability of our findings to geographically diverse populations. Third, certain clinically relevant variables did not included in the present analysis. PD-L1 expression was not available due to a high proportion of missing data, which prevented evaluation of its relationship with the CALI model and its potential interaction with immunotherapy response. Similarly, frailty status and comprehensive geriatric assessment measures were not part of routine clinical documentation at the participating centers during the study period, and could therefore not be retrospectively collected.

## Conclusion

The CALI model offers a simple, bedside-applicable prognostic tool for risk stratification in older patients (≥75 years) with advanced NSCLC receiving immunotherapy. By integrating comorbidity burden, recent weight loss, and inflammatory-nutritional status, the model provides modest but significant incremental prognostic information for OS, though its value for PFS is less consistent. Its performance was validated in independent external cohorts, supporting its potential to facilitate individualized risk stratification and treatment decision-making in this understudied population. However, future prospective studies with larger, more diverse cohorts and inclusion of geriatric assessment domains are warranted to further validate and refine the model.

## Data Availability

The original contributions presented in the study are included in the article/**Supplementary Material**. Further inquiries can be directed to the corresponding author.
