# Supplementary material for: A comprehensive prognostic model for older patients with advanced non-small cell lung cancer receiving immunotherapy: a multicenter real-world study
Source: Front Immunol. 2026 Aug 11;17:1917659. doi: 10.3389/fimmu.2026.1917659 (PMC13503542; doi:10.3389/fimmu.2026.1917659)
Supplement: Supplementary file 1 [file Table1.docx]

**Supplementary Figure Legends:
Supplementary Figure S1. Kaplan-Meier survival curves for conventional indices using median cutoffs in the development cohort.**

(A–H) OS and PFS stratified by median cutoff values for ALI (A, B), PNI (C, D), NLR (E, F), and PLR (G, H).

**Supplementary Figure S2. Kaplan-Meier survival curves for conventional indices using optimal cutoffs in the development cohort.**

(A–H) OS and PFS stratified by optimal cutoff values for ALI (A, B), PNI (C, D), NLR (E, F), and PLR (G, H).

**Supplementary Figure S3. Univariate and multivariate Cox regression analyses for OS in the development cohort.**

Forest plot showing HRs with 95% CIs for baseline clinical variables, comorbidities, inflammatory-nutritional indices, and laboratory parameters in relation to OS.

**Supplementary Figure S4. Univariate and multivariate Cox regression analyses for PFS in the development cohort.**

Forest plot showing HRs with 95% CIs for baseline clinical variables, comorbidities, inflammatory-nutritional indices, and laboratory parameters in relation to PFS.

**Supplementary Figure S5. Time-dependent discriminatory performance of CALI and conventional indices.**

Inverse probability of censoring-weighted cumulative/dynamic AUC curves for CALI, ALI, PNI, NLR, and PLR from 6 to 24 months are presented for OS in the development cohort (A) and pooled validation cohort (B), and for PFS in the development cohort (C) and pooled validation cohort (D). The shaded area represents the pointwise 95% confidence interval for CALI based on 1,000 bootstrap resamples. The horizontal dashed line denotes an AUC of 0.5.

**Supplementary Figure S6. Calibration plots of the CALI model for OS and PFS.**

(A) OS in development cohort A. (B) PFS in development cohort A. (C) OS in the pooled validation cohort B+C. (D) PFS in the pooled validation cohort B+C. Error bars indicate 95% confidence intervals.

**Supplementary Tables:**

**Supplementary Table S1. Parameters used for CALI risk-score calculation in the development cohort.**

| **Variable** | **Coding** | **Distribution** | **μ** | **σ** | **β for OS** | **β for PFS** |
| --- | --- | --- | --- | --- | --- | --- |
| ALI | Continuous | 307.5 ± 190.2 | 307.531 | 190.167 | -0.066990 | -0.012879 |
| Hypertension | 0 = no, 1 = yes | 45 (39.1%) | 0.391 | 0.488 | 0.167307 | 0.087371 |
| Diabetes mellitus | 0 = no, 1 = yes | 21 (18.3%) | 0.183 | 0.386 | 0.078970 | 0.064494 |
| COPD | 0 = no, 1 = yes | 64 (55.7%) | 0.557 | 0.497 | 0.113457 | 0.048055 |
| Weight loss ≥5% | 0 = no, 1 = yes | 26 (22.6%) | 0.226 | 0.418 | 0.126074 | 0.073140 |

**Abbreviations: ALI, advanced lung cancer inflammation index; COPD, chronic obstructive pulmonary disease ;** β represents the Cox regression coefficient used for CALI risk-score calculation based on standardized predictors; μ and σ represent the mean and standard deviation used for z-score transformation in the development cohort.

**Notes:** Risk-score calculation: Z = (x − μ)/σ; linear predictor = ΣβZ; CALI risk score = exp(linear predictor).

The endpoint-specific L2 penalties were λ = 0.5 for OS and λ = 1.0 for PFS.

The development-cohort median risk-score cutoffs were 0.950624 for OS and 0.980752 for PFS.

**Supplementary Table S2. Events per variable and bootstrap internal validation of the CALI model in the development cohort**

| **Endpoint** | **N** | **Events** | **EPV** | **Apparent C-index** | **Mean optimism** | **Corrected C-index (95% CI)** | **Apparent calibration slope** | **Mean slope optimism** | **Corrected calibration slope (95% CI)** |
| --- | --- | --- | --- | --- | --- | --- | --- | --- | --- |
| OS | 115 | 47 | 9.4 | 0.673 | 0.023 | 0.649 (0.561–0.741) | 1.792 | 0.290 | 1.502 (0.643–2.467) |
| PFS | 115 | 69 | 13.8 | 0.609 | 0.030 | 0.578 (0.508–0.660) | 2.125 | 0.554 | 1.572 (0.349–2.870) |

****Abbreviations:** N, number; EPV, events per variable; CI, confidence interval; OS, overall survival; PFS, progression-free survival.**

**Supplementary Table S3. AUC comparisons at 1 and 2 years**

| **Cohort** | **Endpoint** | **Horizon** | **Comparator** | **CALI AUC (95% CI)** | **Comparator AUC (95% CI)** | **Difference (95% CI)** | **P value** | **FDR- adjusted P** |
| --- | --- | --- | --- | --- | --- | --- | --- | --- |
| A | OS | 1-year | ALI | 0.587 (0.455–0.718) | 0.519 (0.386–0.659) | 0.068 (-0.073 to 0.213) | 0.314 | 0.314 |
| A | OS | 1-year | PNI | 0.587 (0.455–0.718) | 0.495 (0.348–0.637) | 0.092 (-0.077 to 0.268) | 0.286 | 0.314 |
| A | OS | 1-year | NLR | 0.587 (0.455–0.718) | 0.505 (0.373–0.644) | 0.082 (-0.065 to 0.233) | 0.246 | 0.314 |
| A | OS | 1-year | PLR | 0.587 (0.455–0.718) | 0.443 (0.295–0.580) | 0.144 (-0.047 to 0.338) | 0.114 | 0.314 |
| A | OS | 2-year | ALI | 0.712 (0.515–0.864) | 0.644 (0.451–0.821) | 0.068 (-0.142 to 0.268) | 0.492 | 0.655 |
| A | OS | 2-year | PNI | 0.712 (0.515–0.864) | 0.691 (0.514–0.848) | 0.020 (-0.230 to 0.239) | 0.871 | 0.871 |
| A | OS | 2-year | NLR | 0.712 (0.515–0.864) | 0.595 (0.402–0.769) | 0.117 (-0.112 to 0.338) | 0.304 | 0.607 |
| A | OS | 2-year | PLR | 0.712 (0.515–0.864) | 0.593 (0.420–0.758) | 0.119 (-0.104 to 0.338) | 0.296 | 0.607 |
| A | PFS | 1-year | ALI | 0.584 (0.442–0.713) | 0.534 (0.401–0.658) | 0.050 (-0.083 to 0.191) | 0.525 | 0.525 |
| A | PFS | 1-year | PNI | 0.584 (0.442–0.713) | 0.505 (0.379–0.636) | 0.079 (-0.089 to 0.239) | 0.362 | 0.525 |
| A | PFS | 1-year | NLR | 0.584 (0.442–0.713) | 0.523 (0.395–0.653) | 0.061 (-0.085 to 0.214) | 0.418 | 0.525 |
| A | PFS | 1-year | PLR | 0.584 (0.442–0.713) | 0.520 (0.382–0.641) | 0.064 (-0.095 to 0.231) | 0.420 | 0.525 |
| A | PFS | 2-year | ALI | 0.708 (0.506–0.895) | 0.634 (0.406–0.829) | 0.074 (-0.150 to 0.306) | 0.531 | 0.709 |
| A | PFS | 2-year | PNI | 0.708 (0.506–0.895) | 0.683 (0.475–0.856) | 0.025 (-0.202 to 0.246) | 0.841 | 0.841 |
| A | PFS | 2-year | NLR | 0.708 (0.506–0.895) | 0.581 (0.372–0.778) | 0.127 (-0.108 to 0.367) | 0.318 | 0.635 |
| A | PFS | 2-year | PLR | 0.708 (0.506–0.895) | 0.557 (0.365–0.742) | 0.151 (-0.105 to 0.398) | 0.242 | 0.635 |
| B+C | OS | 1-year | ALI | 0.561 (0.430–0.690) | 0.501 (0.373–0.636) | 0.060 (-0.088 to 0.197) | 0.400 | 0.706 |
| B+C | OS | 1-year | PNI | 0.561 (0.430–0.690) | 0.557 (0.435–0.679) | 0.004 (-0.170 to 0.167) | 0.973 | 0.973 |
| B+C | OS | 1-year | NLR | 0.561 (0.430–0.690) | 0.495 (0.362–0.635) | 0.067 (-0.096 to 0.217) | 0.404 | 0.706 |
| B+C | OS | 1-year | PLR | 0.561 (0.430–0.690) | 0.499 (0.376–0.629) | 0.062 (-0.131 to 0.241) | 0.529 | 0.706 |
| B+C | OS | 2-year | ALI | 0.806 (0.675–0.916) | 0.589 (0.408–0.759) | 0.217 (0.059 to 0.394) | 0.008 | 0.011 |
| B+C | OS | 2-year | PNI | 0.806 (0.675–0.916) | 0.569 (0.383–0.743) | 0.237 (0.051 to 0.436) | 0.016 | 0.016 |
| B+C | OS | 2-year | NLR | 0.806 (0.675–0.916) | 0.528 (0.360–0.697) | 0.278 (0.095 to 0.453) | 0.004 | 0.011 |
| B+C | OS | 2-year | PLR | 0.806 (0.675–0.916) | 0.412 (0.237–0.606) | 0.395 (0.149 to 0.625) | 0.006 | 0.011 |
| B+C | PFS | 1-year | ALI | 0.553 (0.424–0.685) | 0.539 (0.411–0.663) | 0.013 (-0.140 to 0.186) | 0.901 | 0.911 |
| B+C | PFS | 1-year | PNI | 0.553 (0.424–0.685) | 0.596 (0.458–0.713) | -0.043(-0.208 to 0.133) | 0.667 | 0.911 |
| B+C | PFS | 1-year | NLR | 0.553 (0.424–0.685) | 0.535 (0.409–0.653) | 0.017 (-0.144 to 0.196) | 0.853 | 0.911 |
| B+C | PFS | 1-year | PLR | 0.553 (0.424–0.685) | 0.540 (0.411–0.662) | 0.013 (-0.163 to 0.220) | 0.911 | 0.911 |
| B+C | PFS | 2-year | ALI | 0.793 (0.654–0.927) | 0.520 (0.340–0.704) | 0.273 (0.070 to 0.475) | 0.010 | 0.020 |
| B+C | PFS | 2-year | PNI | 0.793 (0.654–0.927) | 0.624 (0.452–0.795) | 0.168 (-0.039 to 0.392) | 0.118 | 0.118 |
| B+C | PFS | 2-year | NLR | 0.793 (0.654–0.927) | 0.436 (0.278–0.605) | 0.357 (0.162 to 0.555) | 0.002 | 0.008 |
| B+C | PFS | 2-year | PLR | 0.793 (0.654–0.927) | 0.414 (0.168–0.663) | 0.379 (0.044 to 0.692) | 0.024 | 0.032 |

****Abbreviations:** ALI, advanced lung cancer inflammation index; AUC, area under the curve; CALI, comorbidity-ALI model; CI, confidence interval; FDR, false discovery rate; NLR, neutrophil-to-lymphocyte ratio; OS, overall survival; PFS, progression-free survival; PLR, platelet-to-lymphocyte ratio; PNI, prognostic nutritional index.**

**Supplementary Table S4. Formal paired comparisons of the discriminative performance of CALI and conventional indices**

| **Cohort** | **Endpoint** | **Comparator** | **CALI C-index (95% CI)** | **Comparator C-index (95% CI)** | **Difference (95% CI)** | **P value** | **FDR- adjusted P** |
| --- | --- | --- | --- | --- | --- | --- | --- |
| A | OS | ALI | 0.673 (0.566-0.769) | 0.526 (0.421-0.624) | 0.146 (0.036 to 0.264) | 0.020 | 0.020 |
| A | OS | PNI | 0.673 (0.566-0.769) | 0.525 (0.411-0.633) | 0.148 (0.023 to 0.279) | 0.018 | 0.020 |
| A | OS | NLR | 0.673 (0.566-0.769) | 0.506 (0.393-0.608) | 0.167 (0.062 to 0.284) | 0.006 | 0.012 |
| A | OS | PLR | 0.673 (0.566-0.769) | 0.486 (0.391-0.584) | 0.187 (0.064 to 0.320) | 0.006 | 0.012 |
| A | PFS | ALI | 0.609 (0.515-0.691) | 0.503 (0.414-0.586) | 0.106 (0.012 to 0.224) | 0.024 | 0.032 |
| A | PFS | PNI | 0.609 (0.515-0.691) | 0.529 (0.438-0.620) | 0.079 (-0.034 to 0.188) | 0.128 | 0.128 |
| A | PFS | NLR | 0.609 (0.515-0.691) | 0.482 (0.398-0.567) | 0.126 (0.021 to 0.227) | 0.020 | 0.032 |
| A | PFS | PLR | 0.609 (0.515-0.691) | 0.483 (0.394-0.575) | 0.126 (0.020 to 0.232) | 0.016 | 0.032 |
| B+C | OS | ALI | 0.671 (0.580-0.765) | 0.537 (0.434-0.637) | 0.134 (0.016 to 0.259) | 0.022 | 0.022 |
| B+C | OS | PNI | 0.671 (0.580-0.765) | 0.519 (0.421-0.614) | 0.152 (0.033 to 0.267) | 0.008 | 0.016 |
| B+C | OS | NLR | 0.671 (0.580-0.765) | 0.529 (0.431-0.635) | 0.143 (0.030 to 0.264) | 0.018 | 0.022 |
| B+C | OS | PLR | 0.671 (0.580-0.765) | 0.451 (0.353-0.557) | 0.221 (0.082 to 0.345) | 0.004 | 0.016 |
| B+C | PFS | ALI | 0.613 (0.529-0.697) | 0.511 (0.415-0.608) | 0.101 (0.005 to 0.213) | 0.040 | 0.053 |
| B+C | PFS | PNI | 0.613 (0.529-0.697) | 0.532 (0.443-0.619) | 0.080 (-0.021 to 0.183) | 0.118 | 0.118 |
| B+C | PFS | NLR | 0.613 (0.529-0.697) | 0.498 (0.410-0.587) | 0.115 (0.011 to 0.216) | 0.034 | 0.053 |
| B+C | PFS | PLR | 0.613 (0.529-0.697) | 0.445 (0.347-0.539) | 0.168 (0.045 to 0.289) | 0.008 | 0.032 |

****Abbreviations:** See Supplementary Table S3.**
